# Fate of Methicillin‐resistant *Staphylococcus aureus* (MRSA) under simulated acidic conditions of the human stomach

**DOI:** 10.1002/fsn3.1698

**Published:** 2020-07-31

**Authors:** Elisa Spinelli, Teresa Requena, Marta Caruso, Antonio Parisi, Loredana Capozzi, Laura Difato, Giovanni Normanno

**Affiliations:** ^1^ Department of Science of Agriculture, Food and the Environment (SAFE) University of Foggia Foggia Italy; ^2^ Research Institute of Food Science CIAL (CSIC‐UAM) Madrid Spain; ^3^ Experimental Zooprophylactic Institute of Apulia and Basilicata Matera Italy; ^4^ Experimental Zooprophylactic Institute of Apulia and Basilicata Putignano Italy

**Keywords:** food safety, gastric survival, MRSA, ST1, ST398

## Abstract

A known amount (10^7^ cfu/ml) of animal origin Methicillin‐resistant *Staphylococcus aureus* (MRSA) ST398/t011/V and of human origin MRSA ST1/t127/IVa strains were individually inoculated into *ricotta* cheese and hamburger samples. The pH of each food matrix was gradually decreased from 6.0 down to 2.0 during a period of about 2 hr, under conditions simulating the mechanical digestion of the human stomach. Afterward, the MRSA strains were recovered by using a MRSA‐specific plating medium. Although both strains showed a certain acidic resistance, they showed different responses at low pH values during the experiment: ST398 survived unharmed during the course of the experiments to the last stage at pH 2 where counts of 6.4 cfu/g for the hamburger and 7.5 log cfu/g for *ricotta* cheese assays were obtained. In contrast, the ST1 population was no longer detectable at pH 3 in the hamburger and at pH 2 in the *ricotta* cheese assays. To the best of our knowledge, this is the first study that investigates the ability of MRSA to overcome the acidic conditions of the human stomach and that adds new evidence that might contribute to expand the scientific knowledge on the significance of MRSA in the food safety debate.

## Graphical abstract

The presence of MRSA in several kinds of food has suggested that it could act as a foodborne pathogen; however, nothing is known about its ability to overcome the acid‐dependent bactericidal barrier of the human stomach. This in vitro study investigated the survival of MRSA inoculated in two foods of animal origin exposed to the simulated acidic conditions of the human stomach and add a piece of new evidence about the significance of MRSA from a food safety perspective.

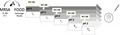

**Highlights**

- The study describes for the first time the fate of human and animal MRSA, inoculated in different food matrices, under simulated acidic conditions of the human stomach.
- The study provides information on the survival of MRSA under the acidic conditions of the human stomach that could be useful for food safety purposes.
- The two strains of MRSA used in this study show different behaviors under the experimental conditions.

## 1 INTRODUCTION

Methicillin‐resistant *Staphylococcus aureus* (MRSA) is a widely known antimicrobial‐resistant bacterium responsible for pathologies, which range from mild to severe, in humans and animals (Doulgeraki, Di Ciccio, Ianieri, & Nychas, 2017). MRSA has been considered almost exclusively a nosocomial pathogen for decades (Doyle, Hartmann, & Lee Wong, 2012); afterward, a great number of studies reported the detection of MRSA in the community, in food‐producing animals and in humans working in close contact with them, such as farmers, veterinarians, and slaughterhouse operators (DeLeo, Otto, Kreiswirth, & Chambers, 2010; Khanna, Friendship, Dewey, & Weese, 2008; Vanderhaeghen, Hermans, Haesebrouck, & Butaye, 2010; Weese, Reid‐Smith, Rousseau, & Avery, 2010), suggesting its zoonotic role (EFSA, 2009). Moreover, both animal and human MRSA strains have been found in several foods of animal origin such as pork (de Boer et al., 2009; O’Brien et al., 2012), poultry (Feβler et al., 2011; Hanson et al., 2011), beef (Tenhagen et al., 2014) and horse meat (Parisi et al., 2017), as well as in raw milk and dairy products (Normanno et al., 2007; Parisi et al., 2016) as a consequence of contaminations occurred during slaughter and milking and, human contamination during food handling. In addition, two food‐related outbreaks (Jones, Kellum, Porter, Bell, & Schaffner, 2002; Kluytmans et al., 1995) and some cases of enterocolitis due to MRSA (Bergevin, Marion, Farber, Golding, & Lévesque, 2017; Pressly, Hill, & Shah, 2016) have been reported. Although the presence of MRSA at different prevalence in foodstuff highlighted its potential as a foodborne pathogen, according to the European Food Safety Authority (EFSA), food contaminated by MRSA is only considered a possible vehicle of transmission (EFSA, 2009). Also, Larsen and colleagues have recently supported the hypothesis that MRSA could be a foodborne zoonotic agent (Larsen et al., 2016); however, to date, there are too many missing pieces to complete the whole puzzle and define MRSA as a foodborne pathogen. In fact, the first requirement for an enteric colonization during an active infection is the ability of bacteria to circumvent the acidic environment of the stomach and pass into the intestinal tract (Gahan & Hill, 2005; Smith, 2003).

It is widely recognized that a normal gastric acidity may provide an important host defense against ingested pathogens by killing these microorganisms, as previously demonstrated for several gram‐negative bacilli and vegetative cells of *Clostridium difficile, Mycobacterium avium*, as well as for nosocomial pathogens, such as *Candida albicans*, Methicillin‐resistant *Staphylococcus aureus* (MRSA), vancomycin‐resistant *Enterococcus* spp. (VRE), and extended‐spectrum‐β‐lactamase‐producing *Enterobacteriaceae* (Bodmer, Miltner, & Bermudez, 2000; Donskey, 2004; Rao, Jump, Pultz, Pultz, & Donskey, 2006).

A number of studies have examined the adaptative responses of *S. aureus* exposed to acid stress in fermented foods (Bore, Langsrud, Langsrud, Rode, & Holck, 2007; Rode et al., 2010). However, little information is available on the ability of acidic conditions of the human stomach to kill ingested MRSA. The aim of this study was to evaluate the survival of two MRSA strains included in two foods of animal origin exposed to the human stomach environment by simulating the gastric acid conditions and its mechanical digestion.

## 2 MATERIAL AND METHODS

### 2.1 Preparation of Methicillin‐resistant *S. aureus* (MRSA) inoculum

A MRSA ST398/t011/V strain, previously isolated from raw cow's milk, and a MRSA ST1/t127/IVa strain, previously isolated from human nasal swabs (Parisi et al., 2016), were individually suspended in 5 ml of BHI broth (pre‐inoculum) and incubated at 37°C for 24 hr. The MRSA culture of each strain was prepared by resuspending, for each one, 20 µl of the pre‐inoculum in 5 ml of fresh BHI broth incubated at 37°C for 24 hr. The MRSA concentration of each inoculum, used in the simulated gastric acidity experiment, was 5 ml × 10^7^ cfu/ml according to the McFarland standard.

### 2.2 Preparation of food matrices

The fed medium was prepared as previously described by Barroso, Cueva, Peláez, Martínez‐Cuesta, and Requena (2015). The medium contained arabinogalactan (1 g/L), pectin from apple (2 g/L), xylan (1 g/L), potato starch (3 g/L), glucose (0.4 g/L), yeast extract (3 g/L), peptone (1 g/L), mucin (4 g/L), and L‐cysteine (0.5 g/L). Once the powders were dissolved, the mixture was then autoclaved, and the pH was adjusted until it reached the value of 6.5.

Two foods of animal origin, *ricotta* cheese and hamburger, were used in the experiments. To exclude bias in the results, each food matrix was tested prior to the experiment for the presence of *S. aureus* and MRSA using the protocol described by Parisi et al. (2016).

For the preparation of the experimental suspension (ES) used in this study, fifty grams of each food matrix were individually added to 50 ml of fed medium at 37°C and homogenized for 10 min at 230 RPM using a stomacher at room temperature. Finally, 5 ml of each MRSA inoculum was added (5% of the volume) to each of the two prepared ES.

### 2.3 Gastric acidity experiment

The simulated gastric acidic experiment was performed as described by Haffner, van de Wiele, and Pasc (2017) with slight modifications. Briefly, the pH of the samples was gradually decreased during a period of about 2 hr and periodically homogenized by using a stomacher miming the mechanical digestion of the stomach (Haffner et al., 2017; Maisanaba et al., 2018). In detail, after the MRSA inoculation into each prepared ES, and its first homogenization (230 RPM for 10 min) in a stomacher bag, the pH of the *ricotta* cheese and the hamburger (stabilized at pH 6.0 starting from pH 6.5 and pH 5.8, respectively) was decreased in steps from 6.0 (T_0_) to 2.0 (T_4_) by adding a specific amount of 1 M HCl (from 0.5 to 19 ml). Each sample was shaken for 2 min at room temperature and incubated at 37°C for 15 min at each point of the experiment (T_1_‐T_3_), except for the last one (T_4_), when they were incubated for 30 min at 37°C, as suggested by Haffner et al. (2017). For each food experiment, 50 ml of fed medium inoculated with each MRSA strain was used as a control. Each control had the same initial pH as its food matrix equivalent, and they were both processed under the above‐mentioned conditions. Each experiment was carried out twice.

### 2.4 MRSA count

Appropriate serial dilutions in peptone water of each sample at each sampling time, from T_0_ (inoculum time) to T_4_ (pH 2), were seeded onto plates of MRSA‐SELECT^®^ (BioRad) and incubated at 37°C for 24 hr. To evaluate the variation in acidic resistance of each MRSA strain during the course of the experiment, at each stage in the decrease of the pH values (from T_0_ to T_4_) in both the matrices and the controls, the coefficient of variation (CV%) was used.

### 2.5 Total bacteria count (TBC)

In order to assess the efficacy of the procedure, the effect of the acidification on the total bacteria of both food matrices used in the study was evaluated. In detail, a total bacterial colony count (TBC) was carried out on both non‐inoculated *ricotta* cheese and hamburger at three different points during the experiment: at T_0_ (pH 6), at T_2_ (pH 4), at T_4_ (pH 2) by using a Plate Count Agar (Microbiol) according to the protocol reported in the ISO 4833–1:^2013^.

## 3 RESULTS

### 3.1 MRSA counts

**FIGURE 1:**
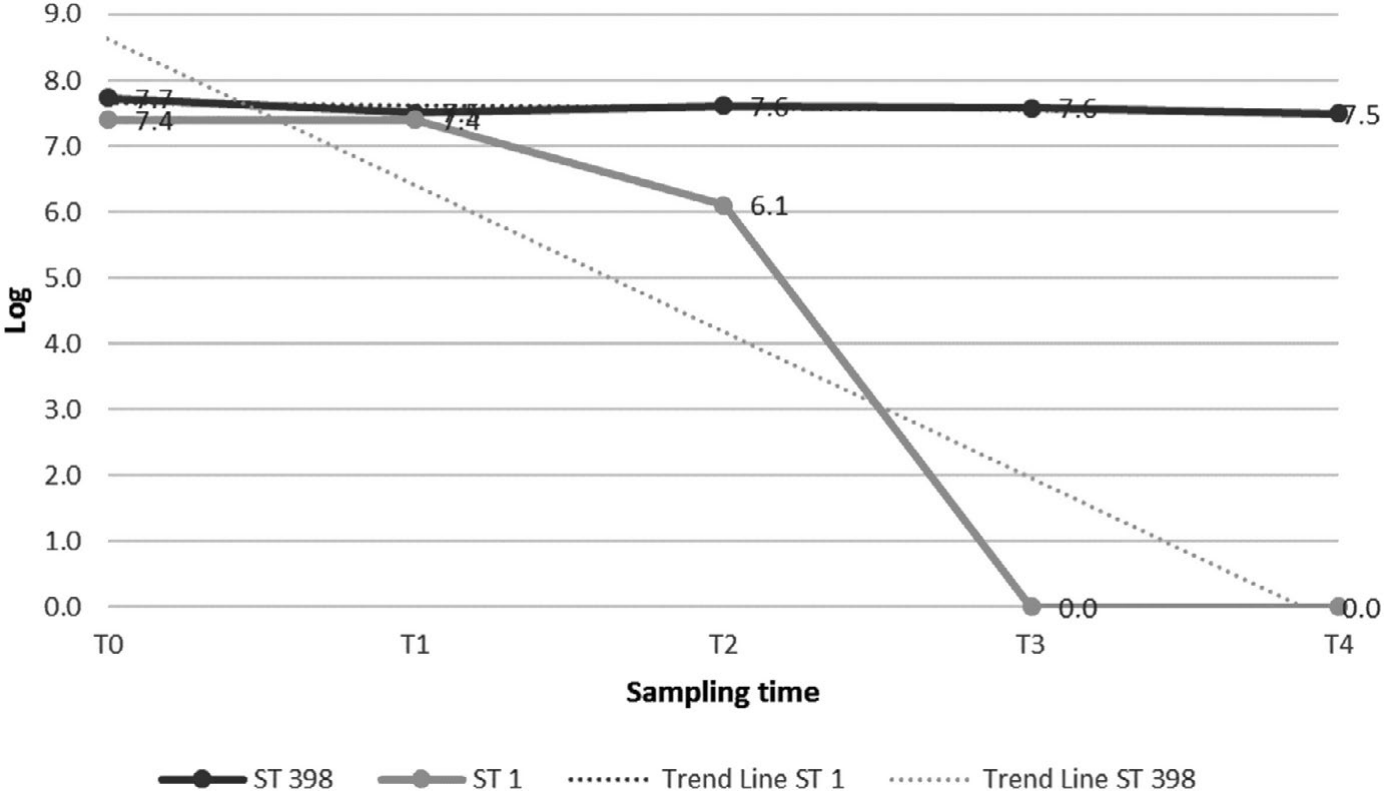
MRSA ST 398 and ST1 counts under acidic stress conditions: control. T_0_ (pH 6) = *inoculum* time; T_1_ (pH 5); T_2_ (pH 4); T_3_ (pH 3); T_4_ (pH 2)

**FIGURE 2:**
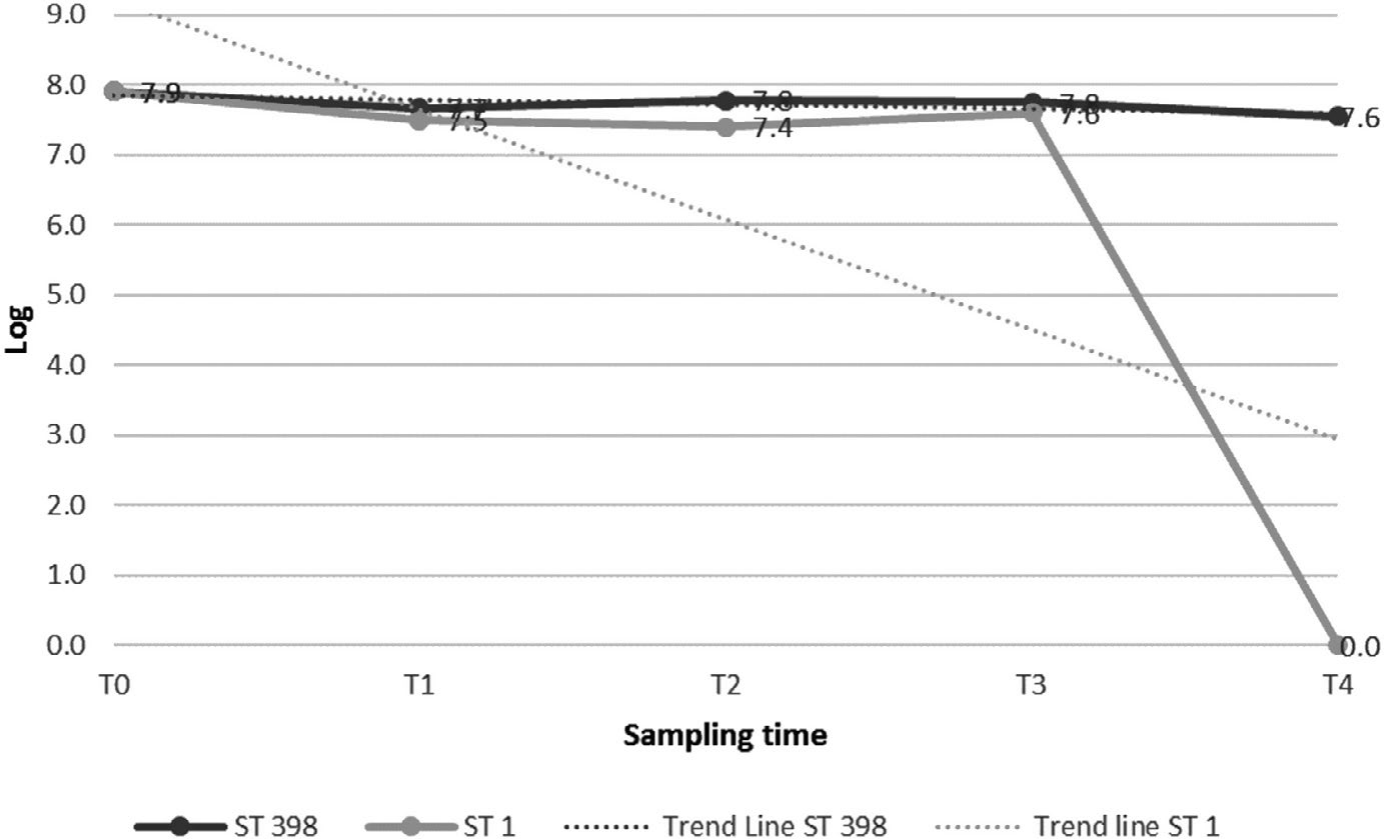
MRSA ST 398 and ST1 counts under acidic stress conditions: ricotta cheese. T_0_ (pH 6) = *inoculum* time; T_1_ (pH 5); T_2_ (pH 4); T_3_ (pH 3); T_4_ (pH 2)

**FIGURE 3:**
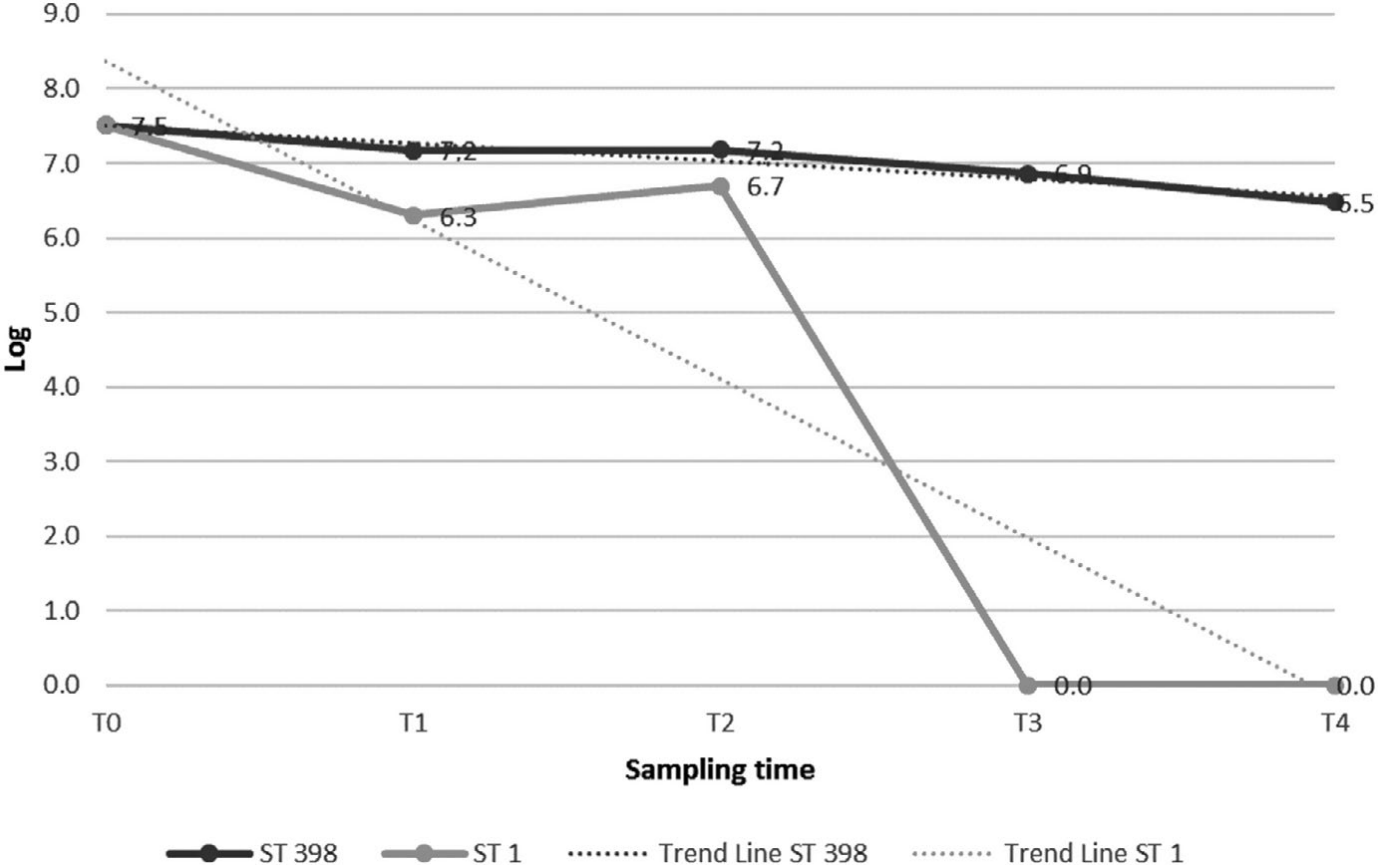
MRSA ST 398 and ST1 counts under acidic stress conditions: hamburger. T_0_ (pH 6) = *inoculum* time; T_1_ (pH 5); T_2_ (pH 4); T_3_ (pH 3); T_4_ (pH 2)

The MRSA counts of both strains showed a decrease during the course of the experiments, as reported in Table 1 and in Figures 1, 2 and 3. The two MRSA strains showed a different variation in acidic resistance at a given pH value. In detail, in the controls and in both the matrices, the variation in acidic resistance of ST398 corresponded to a coefficient of variation under the factor 10, while for ST1 it was over the factor 10^2^.

**TABLE 1:** MRSA ST398 and ST1 counts at each given pH level in the ricotta cheese, hamburger, and control assays

T	pH	Control				*Ricotta* cheese				Hamburger			
		ST 398		ST 1		ST 398		ST 1		ST 398		ST 1	
		Log	R	Log	R	Log	R	Log	R	Log	R	Log	R
T_0_	6	7.7	0.1	7.4	0.4	7.9	0.4	7.9	0.3	7.5	0.4	7.5	0.3
T_1_	5	7.5	0.8	7.4	0.1	7.7	0.5	7.5	0.3	7.2	0.9	6.3	0.3
T_2_	4	7.6	0.2	6.1	0.3	7.8	0.3	7.4	0.2	7.2	0.4	6.7	0.4
T_3_	3	7.6	0.1	0.0	0.0	7.8	0.2	7.6	0.1	6.9	0.2	0.0	0.0
T_4_	2	7.5	0.3	0.0	0.0	7.6	0.2	0.0	0.0	6.5	0.0	0.0	0.0

Note

R = range (of variation) between the two repetitions of each assay.

The survival threshold of both strains was higher in the *ricotta* cheese than into the hamburger (Figures 1, 2 and 3). Counts of log 7.5 and 7.6 cfu/g of ST398 were still obtained at T_4_ (pH 2) in the controls and in the *ricotta* cheese, respectively. The most significant decrease for ST398 was recorded at T_3_ (pH 3) in the hamburger experiment, in which we recorded one log cfu/g less than its concentration at its initial pH (T_0_; pH 6.0) (Table 1). Similarly, the ST1 strain in the *ricotta* cheese was detectable with slight decreases during the course of the experiment until T_3_ (pH 3), when a count of 7.6 cfu/g was still obtained. After that, it was no longer detectable (T_4_; pH 2.0). In contrast, in the hamburgers there was a significant decrease, with a count of log 6.5 cfu/g, at T_2_ (pH 4), after which it was no longer detectable (Table 1).

### 3.2 Total bacteria counts

total bacterial counts showed a decrease during the course of the experiment. In detail, at T_0_ the total bacterial counts, in the *ricotta* cheese and the hamburger experiment, ranged between log 7.4 and 7.0 cfu/g, respectively. At T_2_ (pH 4), the total bacterial count, in both matrices, kept the same values with a light increment for the *ricotta* cheese (log 7.6 cfu/g) and a slight decrease for the hamburger (log 6.9 cfu/g). At T_4_ (pH 2), total bacterial counts were no longer detectable in either of the matrices.

## 4 DISCUSSION

*Staphylococcus aureus* and its methicillin‐resistant variant (MRSA) are microorganisms that have a great impact on both human and veterinary medicine (WHO, 2014). The marked staphylococcal adaptability and its coevolution with its host(s) enable it to be successful as an opportunistic pathogen and to be resistant to changing environments (Clements & Foster, 1998). *S. aureus* is also one of the major foodborne pathogens, representing the leading source of foodborne intoxication (Fetsch & Johler, 2018; Le Loir, Baron, & Gautier, 2003). In addition, MRSA has been identified as an important cause of enterocolitis especially in hospitalized patients and in those who have a decreased gastric acidic production (Pressly et al., 2016). The detection of MRSA in a variety of foods of animal origin, as a consequence of animal and/or human contamination (Normanno et al., 2007), launched a scientific debate on its role in causing infections *via* food consumption, but the survival of MRSA in the acidic conditions of the human stomach has not yet been investigated.

Assuming that the gastric bactericidal barrier is primarily acid‐dependent (Drasar, Shiner, & McLeod, 1969; Hornick et al., 1971; Peterson, Mackowiak, Barnett, Marling‐Cason, & Haley, 1989) because the low pH is able to control bacterial population in the gastric environment (Smith, 2003), we investigated the ability of MRSA to overcome the human gastric barrier by miming the acidic conditions of the stomach.

Considering that MRSA has been detected in raw food, especially meats and cheeses, in our experiments, we simulated a contamination of hamburger and *ricotta* cheese samples with animal (ST398) and human (ST1) MRSA strains in order to evaluate their fate under the acidic environment of the human stomach. We have chosen the ST398 strain for its zoonotic ability (Van den Eede et al., 2013) and the ST1 strain because it is known to be a human pathogen (Monaco et al., 2013). A long‐term mechanical homogenization of each matrix was performed in order to recreate the conditions in which food arrives (as *bolus*) in the proximal part of the stomach after the oral chewing and the transit through the esophagus (Kong & Singh, 2008). According to Haffner et al. (2017) and on the basis of the human gastric digestive phases, the MRSA population was thus exposed to a decreasing pH for different incubation times right down to the last step (T_4_; pH = 2), when the time of exposure to the lower acidic environment was doubled (from 15 to 30 min). In fact, solid foods initially remain in the proximal part of the stomach while liquids pass into the duodenum (Pal, Brasseur, & Abrahamsson, 2007). In a second phase, food particles are mixed, pumped out of the *atrium* and moved from the fundus to the duodenum by propelling actions (Kong & Singh, 2008). MRSA ST1 showed the same trend of TBC, with a dramatic reduction during the course of the experiments, which confirms the efficacy of the acidification process. On the contrary, MRSA ST398 survived unharmed during the course of the entire experiment with a slight decrease from the higher permissive pH value (pH = 6.0) to the final stage at pH 2.

Although not strictly comparable, these results could be explained by what previously found by other authors on the pH stress resistance of *S. aureus*. For example, Chan and colleagues reported that *S. aureus* is rapidly killed by acid (pH 2) but it is able to resist and adapt to acidic stress if it is first exposed to a higher, non‐lethal pH (Chan, Foster, Ingham, & Clements, 1998; Kim et al., 2019; Smith, 2003). Moreover, Rao and colleagues observed a significant in vitro killing of human MRSA exposed to the lower gastric acidic condition (Rao et al., 2006). On the other hand, a study on acidic stress induced by both non‐permeant inorganic acid (HCl) and weak‐permeant organic acid showed that *S. aureus* was affected the most by the organic acid, at the same pH (Lund & Eklund, 2000). Non‐permeant acids do not affect the pH of the cytoplasm as much as weak permeable acids, and microorganisms are generally more sensitive to the internal pH modification than to a change of the external pH (Beales, 2004), confirming the role of the low‐pH‐control in reducing or inhibiting the growth of certain bacteria in food (Rode et al., 2010). The MRSA ST1 strain showed a different acidic resistance under the lower pH in both the matrices and their relative controls. This behavior could be explained by the different composition of the food matrices. In fact, it is well‐known that the food safety involves several intrinsic parameters of food matrices, such as moisture, their activity water (aw), the pH level, the presence of certain preservatives and the microbial ecology (Cho, Lee, & Hwang, 2019). Among these, food nutrients, especially the fat content, play an important role in protecting microorganisms against the acidic stress (Drouault, Corthier, Ehrlich, & Renault, 1999). However, although the hamburger used in this study had a fat content (16%) higher than the *ricotta* cheese (11.6%), the ST1 population showed less acidic resistance in the hamburger experiment, where it was not detectable at the T_3_ (pH 3) than in the *ricotta* cheese experiment, where it was significantly affected by the low pH at T_4_ (pH 2) (Table 1). Further studies need to be carried out in order to explain this behavior as well as the behavioral difference between *S. aureus* and MRSA strains, and between the different MRSA strains under acidic conditions. Moreover, more studies are needed to assess the role of the *mec*A and other antimicrobial‐resistance genes in this finding.

To the best of our knowledge, this is the first study which investigates the behavior of MRSA strains in the acidic conditions of the human stomach. Although we detected differences between the acidic resistance of the two MRSA strains used in our experiments, our results demonstrate that certain strains of MRSA have a strong (prob)ability of surviving under acidic stress conditions. As a consequence, they could pass the gastric barrier and reach the bowel where they could cause an active infection (Bergevin et al., 2017; Pressly et al., 2016; Watanabe et al., 2001).

In conclusion, our results provide new knowledge regarding the fate of MRSA in the acidic conditions of an in vitro human stomach and may contribute to better defining the MRSA role in the food safety debate.

## CONFLICT OF INTEREST

The authors have no conflict of interest to declare.

## ENDNOTE

Published with a contribution from 5 x 1000 IRPEF funds in favour of University of Foggia, in memory of Gianluca Montel.
